# A Prospective Exploratory Study on Potential Systemic Causes and Risk Factors for Subconjunctival Bleeding in Neonatal Calves

**DOI:** 10.3390/vetsci12121111

**Published:** 2025-11-21

**Authors:** Martin Steffl, Katharina Euchner, Nadine Nautscher

**Affiliations:** Institute of Animal Science, University of Hohenheim, 70593 Stuttgart, Germany; katharina.euchner@uni-hohenheim.de (K.E.); n.nautscher@uni-hohenheim.de (N.N.)

**Keywords:** subconjunctival bleedings, neonates, calves, hematology, neutrophils:lymphocytes ratio, LOXL, ELISA

## Abstract

Subconjunctival bleeding in neonatal calves is most commonly seen in association with birth trauma. There are currently no investigations available that examine the systemic causes of this phenomenon. In this prospective and exploratory case–control study, seven out of eighty neonatal calves examined over a two-year period were born with subconjunctival bleeding. The anatomical location of the subconjunctival bleeding and details related to the cow’s and calf’s parturitional and gestational history were recorded. Blood samples from cases and controls (*n* = 7) were analyzed hematologically, and the serum lysyl oxidase-like enzyme 4 (LOXL4) concentration was determined through an ELISA to establish evidence for possible structural, copper-dependent vascular abnormalities. We found no significant difference in the clinical data of both groups. Hematological examinations revealed no evidence of anemia or thrombocytopenia. Additionally, no significant differences in differential leukocyte counts were observed between the different groups. However, the neutrophil–lymphocyte ratio (NLR) demonstrated a significant difference between the calves with subconjunctival bleeding and controls. The serum LOXL4 protein concentration was not significantly different in calves with subconjunctival bleeding compared to controls. In conclusion, our clinical, hematological, and biochemical data provided no evidence for potential systemic causes of subconjunctival bleeding. However, these results must be considered in light of this study’s small sample size and thus low statistical power.

## Simple Summary

To date, subconjunctival bleeding in neonatal animals has not been systematically investigated. In most cases, injuries related to birth have been suspected. This prospective and exploratory case–control study aimed to identify some potential systemic causes and risk factors in neonatal calves (seven animals in each group). Since copper deficiency during pregnancy can have a negative effect on vascular stability, the copper-dependent enzyme LOXL4 was measured in serum. However, our clinical, hematological, and biochemical (LOXL4) examinations did not reveal any evidence of a systemic cause. Further studies with larger numbers of animals could be helpful in the future.

## 1. Introduction

Subconjunctival bleeding is characterized by the appearance of a sharply circumscribed redness caused by bleeding under the bulbar conjunctiva in the absence of inflammatory signs or pathological vessels [1]. Major causes of subconjunctival bleeding can be classified into ocular and systemic conditions [2,3]. In large neonatal animals, subconjunctival bleeding most commonly originates from direct trauma associated with the birth event [4,5]. Systemic conditions that may lead to subconjunctival bleeding in animals potentially include systemic vascular diseases, sudden severe venous congestion in the head, and blood dyscrasia associated with thrombocytopenia and platelet dysfunction [2]. However, there are currently no investigations available that examine systemic causes of subconjunctival bleeding in neonatal animals. In our previous study, all calves with subconjunctival bleeding were born without obvious birth difficulties [6], which raises doubts regarding the preferred trauma theory. In our prospective case–control study, we aimed to investigate systemic conditions leading to subconjunctival bleeding. Blood samples were collected and analyzed hematologically to rule out disorders including anemia and thrombocytopenia. Furthermore, we focused on copper (Cu) metabolism because Cu serves as a cofactor for lysyl oxidase (LOX) and lysyl oxidase-like (LOXL) enzymes, which are critical for the stability of connective and vascular tissues [7]. Based on the similarity of the N-terminal region, copper-dependent LOX enzymes can be divided into two groups: the first group includes LOX and LOXL1 and the second group includes LOXL2, LOXL3, and LOXL4 [7]. LOX and LOXL1 isoforms are preferentially involved in the covalent crosslinking of collagen and elastin in the extracellular space [8]. Within the second group, LOXL2 and LOXL4 have been reported to perform collagen IV crosslinking activities in endothelial basement membranes [9,10].

Cu deficiency during pregnancy can result in several structural abnormalities, including vascular fragility as a result of weak LOX activity [11]. However, Cu and ceruloplasmin are not reliable indicators of the Cu status for neonatal ruminants because neonatal serum Cu and ceruloplasmin concentrations are low and represent only about 20% of the maternal concentrations [12]. Since LOXL4 is extracellularly secreted [10] and is particularly prominent in ocular vessel walls [13]; additionally, we determined the LOXL4 protein concentration in the serum of calves with and without subconjunctival bleeding. Our hypothesis was that calves with subconjunctival bleeding may have lower serum LOXL4 concentrations than control calves, most likely indicating Cu-dependent vascular abnormalities.

## 2. Materials and Methods

### 2.1. Animals

Examinations were performed at the experimental station for Agricultural Sciences of the University of Hohenheim over a two-year period from November 2021 to October 2023. On average, 45 Holstein Friesian cows and a few Jersey dairy cattle (data extracted from the dairy quality control records, LKV Baden-Württemberg, Stuttgart, Germany) were kept in a pen with a separate calving barn. Living as a stable herd, individual cows that were heavily pregnant were moved to the separate calving barn approximately one week before their calculated calving date. The separate calving barn had space for a maximum of four cows in spacious individual stalls. Dry cows were fed a specially formulated total mixed ration (TMR) consisting of corn silage, grass silage, hay, barley straw, and various additives. Calving was only monitored if it took place between 4 a.m. and 7 p.m. In some cases, calving also took place at night without supervision.

Within a few hours to a few days after birth, all calves were thoroughly examined by experienced veterinary surgeons according to Good Veterinary Practice (GVP). The occurrence and location of subconjunctival bleeding and details of the cow’s and calf’s parturitional and gestational history were recorded (Table 1 and Table 2). Calving difficulty was scored according to Barrier and Haskell [14]. Cases and controls were recruited prospectively. Blood samples from control calves were only collected when calves were in good health and if there was no evidence of dystocia. For better comparability, whenever possible, blood samples were collected on the same day as those of the affected calves. Seven out of eighty neonatal calves examined over the two-year period were born with subconjunctival bleeding. Additionally, seven control calves were included in this study. In nine cases, the calves were one day old or younger. The other five calves were between two and five days old (Table 1). The majority of these calves were control animals because they were usually born around a similar time to a calf with bleeding.

### 2.2. Hematological Analysis

Blood samples from jugular veins were collected chronologically from November 2021 (calf no. 1) to October 2023 (calf no. 14). In five out of seven cases, blood samples from control calves were collected on the same day as the blood samples of affected animals. All EDTA-anticoagulated samples were analyzed within one hour after collection. Two freshly prepared blood smears were stained with a May–Grünwald and Giemsa solution. Hematological analyses were performed using an automated animal blood counter (scil Vet abc Plus+, Scil animal care company, Viernheim, Germany). This impedance-based hematology instrument was found to produce acceptable results for in-clinic use [15]. Differential leucocyte counts for 200 cells were performed on each slide at a magnification of 1000× using the battlement counting technique [16]. A total of 400 cells were identified on two slides from each blood specimen. The number of each cell type was recorded, and the results were expressed as percentages of the total count. Afterwards, the percentage of each present leukocyte type was multiplied by the total leukocyte count to obtain the absolute number of each cell type per mm^3^ [17]. Platelet counts were determined using the scil Vet abc Plus+ analyzer. Results deviating from the reference range were confirmed through a microscopic examination of the blood smear. The average number of platelets in 10 oil immersion fields was multiplied by 15,000 to yield a platelet count estimate per mm^3^. For all parameters, the average value and standard deviation were calculated from seven measurements for both the cases and the controls.

### 2.3. Determination of Lysyl Oxidase 4 (LOXL4) Serum Protein Concentration

Serum was prepared from whole blood samples in plain tubes immediately after collection and were frozen at −20 °C until processing. The determination of the lysyl oxidase 4 (LOXL4) concentration in serum samples was performed five months after the last sampling in October 2023 using the bovine lysyl oxidase homolog 4 (LOXL4) ELISA Kit (No. G-BOEB0209.96, Biomol, Hamburg, Germany). According to the manufacturer, Assay Genie, this ELISA Kit has been validated in bovine serum samples diluted 1:5.

Laboratory technicians were blinded to group assignments during blood analysis. The standard curve for LOXL4 ranged from 0 to 5 ng/mL. The intra- and inter-assay coefficient of variation (CV) values were 1.98 and 12.58%, respectively. The LOX4 concentration in the blood was determined twice for each sample, and an average value was calculated. An average value plus the standard deviation was determined for both the case and control groups from all average values.

### 2.4. Statistical Analysis

Hematological traits were analyzed using a linear model that fits two separate means for each group. The model was extended by using sex, age, parity, and gestational time as co-variables, if these were significant (*p* < 0.05). For LOXL4, the model was extended by fitting a fixed run effect and random animal effects. Additionally, data were logarithmically transformed prior to analysis. For all traits, tests for differences between groups were considered as significant for $\frac{p}{m}$ < 0.05, where *p* is the *p*-value of the single test and *m* is the number of tests (11) performed in total [18]. Additionally, equivalence tests were performed. In this case, means of both groups were considered as equivalent if the 90% confidence limit of the group difference was completely overlapped by the pre-defined interval [−δ;δ]. All statistical analyses were performed with SAS software (version 9.4, SAS Institute, Cary, NC, USA).

For all variables, the power was calculated using the Stroup’s approach [19], with a non-central F distribution. With this method, we found that the difference and the estimated variance components were fixed, and the power was calculated. The interpretation of the value is as follows: The power is the probability that a true difference as large as the observed difference observed in the study is found as significant if the studies were conducted similarly. Therefore, for the logwert trait a difference of 0.2837 (ratio of 1.328) and a power of 0.16182 were found. If the true difference between the groups is 0.2837, then in about 16% of the studies conducted with the current sample size, such a difference can be determined as significant.

## 3. Results

### 3.1. Anatomical Location of Subconjunctival Bleeding and Associated Factors

Over the two-year period, we identified seven calves with subconjunctival bleeding affecting eight eyes. Five calves were female and two were male. Four calves displayed bleeding in the left eye, two displayed bleeding in the right eye, and one calf was affected bilaterally (Table 2). In all but one of the cases the bleeding occurred dorsally. The dorsal location varied from dorsotemporal to dorsonasal without a clear preference. In most cases, the type of subconjunctival bleeding could be characterized as a stripe or macule of a variable size. In two cases we observed subconjunctival bleeding in a semilunar fashion immediately outside the limbus.

In all cases, the affected and control calves were born without assistance (Table 2). There was no significant difference in the age, sex, gestational time (279 vs. 282 days on average), or parity (1.57 vs. 2.29 days on average) between the affected and control calves and their dams.

Primiparous cows were slightly more associated with calves with subconjunctival bleeding (Table 2). The ratio of primiparous to multiparous cows was four (57.1%) to three (42.9%).

### 3.2. Hematology

Hematological parameters, such as the white blood cells, neutrophils, lymphocytes, red blood cells, hemoglobin, hematocrit, mean corpuscular volume (MCV), mean corpuscular hemoglobin (MCH), mean corpuscular hemoglobin concentration (MCHC), and platelets, did not significantly differ between the affected and control calves (Table 3). However, the neutrophil–lymphocyte ratio (NLR) was significantly higher in calves with subconjunctival bleeding compared to control calves (3.80 vs. 1.27, *p* = 0.0007). Interestingly, in calf no. 6, blood values for hemoglobin (16.6 g/dL), hematocrit (35%), MCV (44 µm^3^), MCH (21 pg), and MCHC (47.4 g/dL) were 7.6% to 69.4% higher, on average, than those of the control calves.

### 3.3. Concentration of LOXL4 Protein

As measured by the ELISA, the LOXL4 protein concentration in the serum of calves with subconjunctival bleeding compared to control calves was not significantly different (0.99 ± 1.11 vs. 0.56 ± 0.09 ng/mL, *p* = 0.3153). In the group of calves with subconjunctival bleeding, there was an animal (calf no. 6) with unusually high serum LOXL4 concentrations (3.50 ± 0.68 ng/mL). Results were confirmed by a second test run (Table 4).

## 4. Discussion

In our previous study, we found that 7 out of 289 neonatal calves (2.4 percent) examined over a six-year period had subconjunctival bleeding [6]. At that time, no evidence of local trauma was found; thus, the present study focused on several possible systemic factors. The clinical data showed no significant differences in terms of age, sex, gestational time, and parity. No difficulties were observed during calving, but not all calvings were monitored, and the duration of the birth was not measured. For this reason, local trauma to the eye globe cannot be completely ruled out. In human patients with traumatic subconjunctival bleeding, the frequency of the bleeding was twice as high in temporal areas compared with nasal areas [20]. One possible reason for this may be that the nose protects the nasal aspect of the eyeball from sources of trauma [20]. However, in our study neonatal calves had subconjunctival bleeding mainly in the dorsal conjunctiva, which agrees with Munroe’s observations in foals [5]. Combined with the fact that all calves were born without assistance, direct trauma to the globe is not likely a major cause of subconjunctival bleeding in neonatal calves.

In human babies, the compression of the thorax and abdomen during delivery may raise the venous pressure in the head and may subsequently cause subconjunctival bleeding [21]. Pressure applied to a calf’s head may be caused by a narrow birth canal, delivery forces, and inappropriate human assistance during delivery [4]. Inappropriate human assistance can be excluded in the present investigation because all calves were born without assistance. Strong delivery-force-related pressures applied to the calf’s head appear to be a likely cause of subconjunctival bleeding. A higher speed and magnitude of delivery forces in multiparous mares have been previously suspected by Munroe [5] as etiological factors in foals with subconjunctival bleeding. However, Munroe [5] also considers a combination of several factors to be possible, including an increase in the size of the fetal head and thorax and strong delivery forces. Pressure sensors placed within the birth canal and video recordings of mostly nocturnal calvings would be helpful in the future to identify causative factors.

Hematological analyses were performed to gain further insight into the pathophysiology of subconjunctival bleeding.

In summary, our hematological examinations revealed no evidence for anemia or thrombocytopenia as systemic factors of subconjunctival bleeding. Additionally, no significant differences in leukocyte, neutrophil, and lymphocyte counts were observed between the case and control groups. The mean leukocyte count in Holstein calves was reported to be 9.87 ± 3.90 × 10^9^/L [22] and is similar to the leukocyte counts in our affected calves (11.21 ± 4.72) and control calves (9.33 ± 2.45).

However, the mean NLR differed significantly between calves with subconjunctival bleeding and control calves (3.80 vs. 1.27, *p* = 0.0007). In a previous study, the mean NLR of 254 Holstein calves from 1 to 9 days old (mean ± SD: 1.68 ± 2.50) [22] was similar to the mean NLR of our control calves (1.27 ± 0.70).

The NLR is considered a sensitive indicator of inflammation and oxidative stress in humans and animals [23,24]. Thus, the NLR integrates information on both inflammatory and stress responses [24]. Increased venous pressure to the head and neck is produced by elevated intrathoracic pressure. Elevated intrathoracic pressure in turn arises from the compression of the fetal abdomen and thorax during uterine contractions [21]. We therefore suggest that events associated with elevated intrathoracic pressure may cause hypoxic conditions. It is known that hypoxic conditions favor increases in reactive oxygen species and oxidative stress, both recognized as important pro-inflammatory mediators [25]. This may explain the increase in the NLR. Similarly, it has been demonstrated that pre-eclampsia, characterized by a low intermittent uterine blood flow to the placenta, leads to fetal hypoxia and oxidative stress [26]. It is not surprising that the NLR was significantly higher in neonates of pre-eclamptic mothers [27]. In summary, increased NLRs in neonatal calves with subconjunctival bleeding may indicate stressful hypoxemic events in utero and during calving. However, other reasons such as infection or cortisol-related increases are also conceivable causes of the elevated NLR.

Cu deficiency during pregnancy is thought to affect embryonic and fetal development through mechanisms such as an altered extracellular matrix (ECM) composition and integrity secondary to decreased LOX activity [11]. As previously mentioned, LOXL2 and LOXL4 conduct collagen IV crosslinking functions in endothelial basement membranes [9,10]. Additionally, it is known that LOXL4 is particularly prominent in ocular vessel walls [13]. Our hypothesis, therefore, was that a reduced activity and/or expression of LOXL4 in endothelial cell membranes due to changes in Cu bioavailability may result in a disruption of collagen IV crosslinking activity. As a result, ocular vessels, probably conjunctival vessels, may demonstrate increased fragility in the presence of specific trigger factors, such as elevated venous pressure in the head. However, the LOXL4 protein concentration in the serum of calves with subconjunctival bleeding was not significantly different from that of the control calves. This result can be discussed from various perspectives. Firstly, it can be concluded that determining LOXL4 protein levels in serum is not helpful for diagnosing Cu deficiency in bovine neonates. It is also possible, however, that we have not determined the correct LOXL isoform because members of the LOX family of proteins are differentially expressed within different tissues [7]. Finally, our findings do not rule out the possibility that fluctuations in Cu availability occurred during pregnancy, causing the walls of the eye vessels to become fragile. This could only be determined by measuring the LOXL activity in the tissue itself. Cu deficiency is known to reduce LOX activity but does not necessarily affect LOX protein production [28].

The unusually high LOXL4 values in calf no. 6 may be explained by hypoxia. It is known that hypoxia increases certain members of the LOX family at both the protein and gene level [29]. It is therefore conceivable that this calf was born in severe hypoxic conditions and that the LOXL4 protein expression, like the LOX protein expression, was upregulated by hypoxia/hypoxia-inducible factors [30]. Our assumption is indirectly supported by the fact that elevated hematological parameters were found in this calf. In calf no. 6, blood values for the hemoglobin, hematocrit, MCV, MCH, and MCHC were 7.6% to 69.4% higher, on average, than those of the control calves. The most important elevations were observed in hemoglobin (69.4%) and MCH (65.4%), which in turn may indicate hypoxia, as it is also known to regulate components of the hemoglobin synthesis pathway [31]. Since we did not measure any other indicators of perinatal asphyxia and oxidative stress, this assumption remains highly speculative.

A major limitation of our study is the small number of animals examined, which limits this study’s statistical power (only 16%). Multi-center collaboration is warranted to achieve an adequate sample size in future research. Additionally, the laboratory methods we used appear to be insufficient to test our hypotheses. We only determined one LOXL isoform (LOXL 4). The determination of additional LOXL isoforms and LOXL activity may be a better strategy for investigating significant relationships. Additionally, the high variability in LOXL4 values may prevent accurate and reliable statistical analysis. Minor limitations are that we did not record the duration of the calving. Differences in the calving duration might help to explain stressful hypoxemic events in utero. Also, some calvings occurred at night without supervision. For this reason, a difficult birth occurring at night, for example, due to a narrow birth canal, could therefore have been overlooked without supervision.

## 5. Conclusions

In this small preliminary study, no definitive systemic cause for subconjunctival bleeding was identified using clinical, hematological, and biochemical (LOXL4) methods. The finding of an elevated NLR warrants further investigation through a larger, more robustly designed study.

## Figures and Tables

**Table 1:** The age and sex of each calf in the survey. All calves were Holstein Friesians.

Calf No.	Age (Days), Sex	Case/Control
1	1, female	Case
2	1, male	Control
3	1, male	Case
4	2, female	Case
5	2, female	Control
6	1, male	Case
7	5, female	Control
8	1, female	Case
9	4, male	Control
10	1, female	Control
11	1, female	Case
12	3, female	Control
13	1, female	Case
14	1, female	Control

**Table 2:** Location of subconjunctival bleeding and details of the cow’s and calf’s parturitional and gestational history.

Calf No.	Location or Control	Gestational Time (Days)	Parity of Dam	Calving Difficulty
1	L: D	274	1	N
2	Control	279	3	N
3	L: D + T	282	3	N
4	L: semilunar (D), R: D + NA	276	2	N
5	Control	280	2	N
6	L: semilunar (D)	285	1	N
7	Control	281	1	N
8	R: T + NA	279	2	N
9	Control	283	2	N
10	Control	291	2	N
11	L: D	278	1	N
12	Control	277	5	N
13	R: D + T	281	1	N
14	Control	283	1	N

L: Left; R: Right; D: Dorsal; NA: Nasal; T: Temporal; and N: No assistance.

**Table 3:** Hematological parameters (mean ± standard deviation) for calves with subconjunctival bleeding (cases) and control calves.

Parameter	Cases	Controls	*p*-Value
WBC (10^3^/mm^3^)	11.21 ± 4.72	9.33 ± 2.45	n.s.
Neutrophils (10^3^/mm^3^)	8.53 ± 3.78	4.80 ± 2.03	n.s.
Lymphocytes (10^3^/mm^3^)	2.40 ± 1.07	4.17 ± 1.66	n.s.
N:L ratio (-)	3.80 ± 1.18	1.27 ± 0.70	0.0007
RBC (10^6^/mm^3^)	7.84 ± 1.12	7.67 ± 0.51	n.s.
HGB (g/dL)	10.66 ± 2.89	9.76 ± 0.75	n.s.
HCT (%)	32.10 ± 5.36	31.26 ± 3.12	n.s.
MCV (µm^3^)	40.71 ± 2.21	40.86 ± 2.54	n.s.
MCH (pg)	13.63 ± 3.28	12.73 ± 0.52	n.s.
MCHC (g/dL)	33.21 ± 6.39	31.31 ± 1.32	n.s.
PLTs (10^3^/mm^3^)	525.6 ± 165.4	550.1 ± 352.3	n.s.

WBC: white blood cell count; N:L ratio: neutrophil–lymphocyte ratio; RBC: red blood cell count; HGB: hemoglobin concentration; HCT: hematocrit; MCV: mean corpuscular volume; MCH: mean corpuscular hemoglobin; MCHC: mean corpuscular hemoglobin concentration; PLTs: platelets; and n.s.: not significant.

**Table 4:** LOXL 4 protein concentration (ng/mL) measured by ELISA in serum of calves with subconjunctival bleeding compared to control calves (mean ± standard deviation (SD)).

Calf No.	Cases	Calf No.	Controls	*p*-Value
1	0.47 ± 0.06	2	0.56 ± 0.00	
3	0.69 ± 0.07	5	0.50 ± 0.08	
4	0.49 ± 0.10	7	0.65 ± 0.11	
6	3.50 ± 0.68	9	0.45 ± 0.07	
8	0.62 ± 0.14	10	0.69 ± 0.16	
11	0.56 ± 0.05	12	0.52 ± 0.11	
13	0.59 ± 0.10	14	0.53 ± 0.02	
Mean ± SD:	0.99 ± 1.11		0.56 ± 0.09	*p* = 0.3153

## Data Availability

The original contributions presented in this study are included in the article. Further inquiries can be directed to the corresponding author(s).
